# A Retrospective Naturalistic Study Comparing the Efficacy of Ketamine and Repetitive Transcranial Magnetic Stimulation for Treatment-Resistant Depression

**DOI:** 10.3389/fpsyt.2021.784830

**Published:** 2022-01-13

**Authors:** Georgios Mikellides, Panayiota Michael, Lilia Psalta, Teresa Schuhmann, Alexander T. Sack

**Affiliations:** ^1^Department of Cognitive Neuroscience, Faculty of Psychology and Neuroscience, Maastricht University, Maastricht, Netherlands; ^2^Cyprus rTMS Centre, Larnaca, Cyprus; ^3^Department of Psychology, University of Cyprus, Nicosia, Cyprus; ^4^School of Science, University of Central Lancashire, Preston, Cyprus; ^5^Department of Psychiatry and Neuropsychology, Brain + Nerve Centre, School for Mental Health and Neuroscience, Maastricht University Medical Centre+, Maastricht, Netherlands

**Keywords:** treatment resistant depression, antidepressants, ketamine, IM ketamine, repetitive transcranial magnetic stimulation, iTBS, DLPFC

## Abstract

Depression is a common mental disorder that affects many people worldwide, while a significant proportion of patients remain non-responsive to antidepressant medications. Alternative treatment options such as ketamine therapy and repetitive transcranial magnetic stimulation (rTMS) therapy are offered nowadays. This study aims to describe and compare the acute antidepressive efficacy of both, intramuscular ketamine and rTMS in depression patients seeking help in a naturalistic clinical mental health setting. The clinical records of 24 patients with treatment resistant depression were collected from the clinical base of a real life clinic. Twelve patients were treated with intramuscular ketamine, twice weekly for 8 sessions, and twelve patients were treated with 30 sessions of left dorsolateral prefrontal cortex – intermittent theta-burst stimulation (DLPFC-iTBS). Using three clinical assessments (HDRS, HAM-A, BDI-II), our data reveal that both therapies led to significant improvement in symptoms from pre- to post- treatment, as well as that the two experimental groups did not differ significantly with respect to pre- to post- depressive and anxiety symptoms, indicating that the effect of both experimental groups in our sample was equally effective. Furthermore, our results showed high remission and response rates in both groups, with no statistical differences between the patients of ketamine group and rTMS group in remission and response rates. We show a significant pre- to post- treatment reduction in depressive and anxiety symptoms, with no significant differences between the two experimental groups, indicating that the effect of both therapies was equally effective in our limited sample.

## Introduction

Depression is a common mental disorder that affects more than 264 million people worldwide, irrespective of age (1). Clinically effective first-line treatments include pharmacotherapy and psychotherapy. However, ~30% of depression patients remain non-responsive to antidepressant medications and are suffering from treatment resistant depression (TRD) (2, 3). Conventionally, TRD is diagnosed when a patient is not experiencing any significant clinical improvement from at least two different methods of antidepressants (4). TRD patients are therefore in need of new (non)pharmacological treatment alternatives.

In recent years, there has been a considerable interest in the use of ketamine as an antidepressant in humans. Ketamine is a racemic mixture of two enantiomers, S-ketamine (esketamine) and R-ketamine and the antidepressant properties of N-methyl d-aspartate (NMDA) receptor antagonists have received much attention in experimental animal studies several years ago (5, 6). In patients suffering from TRD, the antidepressant effect of ketamine can be observed within a few hours following a single subanesthetic intravenous infusion (7). As reported by a two-site randomized controlled trial, a single infusion of ketamine was associated with greater improvement in the Montgomery-Åsberg Depression Rating Scale (MADRS) score, compared to an active placebo control condition (anesthetic midazolam), 24 h after treatment (7). The administration of ketamine was not only found to be effective for treating depression, but also in bipolar disorder, as well as in suicidal ideation (8, 9). Furthermore, repeated administration of ketamine may be associated with rapid, longer-term and sustained antidepressant effects (10, 11). According to a recent article by Kim and colleagues (10), methyl-CpG-binding protein 2 (MeCP2) phosphorylation at Ser421 (pMeCP2) plays a crucial role in the sustained antidepressant effects of ketamine in mice. The authors also found that repeated ketamine administration induces processes of metaplasticity through post-synaptic functional changes. This may explain why repeated intake of ketamine doses produce sustained effects (10).

As a drug with brief euphoric effects that may last from 1 to 2 h, ketamine must be administrated under controlled settings (12). The most common adverse effects of ketamine administration are dizziness, drowsiness, poor coordination, blurring of vision, feeling strange, light-headedness, headache and nausea (13).

Ketamine is associated with a robust increase in glutamate and dopamine release in the prefrontal cortex as well as with improvement in neuroplasticity within the hippocampus. Both these brain regions play a crucial role in the pathophysiology of depression (14). The first randomized clinical trial (RCT) that aimed to assess the effectiveness of a single dose of an NMDA receptor antagonist in depressed patients showed a robust significant improvement in depressive symptoms within 3 days post ketamine (15). A recent review and meta-analysis highlighted the effectiveness of a single ketamine (0.5 mg/kg) infusion in reducing depression scores in TRD participants (5). The impact of ketamine was found to be rapid, as the antidepressant effect was observed 4 h post-infusion. However, a subsequent reduction of this antidepressant effect of ketamine appeared 7 days post-infusion, so its effectiveness seems to be short-term (5). In line with this, also other studies documented that the antidepressant effect of a single dose of ketamine typically vanishes after ca 7 days (7, 12).

Ketamine can be delivered in several manners such as via intravenous (IV), intranasal, oral, sublingual, subcutaneous and intramuscular (IM) routes (13). Only very few studies are available that investigated the potential use of IM ketamine delivery in the treatment of depression (16–18). A recent study aimed to compare the safety, tolerability, and efficacy of IM and IV ketamine delivery in treating major depression, showing that a small dose of IM ketamine (0.25 mg/kg) is as effective and safe as a larger dose (0.5 mg/kg). No statistically significant differences were found between IM and IV groups. Reduction of HAM-A scores have been reported 2 h post ketamine in all groups and sustained for the following 3 days. The adverse effects were mild and subsided within an hour post ketamine (19). Furthermore, 6 IV ketamine infusions over a 12-day period were associated with a large, sustained effect as the median time to relapse was 18 days (11).

The United States Food and Drug Administration (FDA) approved ketamine (ketalar) for human use for the first time in 1970 and more recently, in 2019, approved esketamine as an intranasal spray for the treatment of TRD in adults who have failed to receive sufficient improvements from other antidepressant medicines (20). However, the intranasal application of ketamine might not be the best treatment for TRD patients. According to a recent systematic review and meta-analysis, esketamine was found to be less effective, compared to racemic IV ketamine, in treating depression (21). IV and IM administrations of ketamine were also found to be 100 and 93% bioavailable, respectively, in contrast to other routes of administration such as intranasal, which is only 8–45% bioavailable (22). Medications with higher bioavailability could potentially be more effective. Furthermore, treatment using intranasal esketamine spray is more expensive compared to treatment using IV or IM ketamine. Ketamine can be safely given through the IM route and has an easier access of administration than then IV route. In the present study, we therefor applied IM ketamine.

Another, fundamentally different treatment alternative for TRD that has received much attention in the literature is transcranial magnetic stimulation (TMS). TMS is a non-invasive brain stimulation method using the repetitive administration of electromagnetic pulses to targeted regions in the brain to modulate neural activity (23). Repetitive TMS (rTMS) has been shown to lead to longer lasting neuroplastic changes with beneficial clinical effects across various neuropsychiatric disorders (24, 25). rTMS is by now a clinically proven effective, widely recognized, approved and well-tolerated depression therapy in TRD patients (26–28).

The dorsolateral prefrontal cortex (DLPFC) is the most prominent and commonly used target area in rTMS treatment of depression (29–32). TMS over the left DLPFC for several weeks has been shown to be a safe and effective treatment for TRD (28), including often reported beneficial effects on psychomotor speed and cognitive control (33). Furthermore, TMS over the left DLPFC is associated with improvements of suicidal ideation in adolescents with depression (34). One of the largest studies testing the effectiveness of rTMS in depression, the THREE-D study, documented clinically meaningful improvements in patient-reported outcomes (PROs), including quality-of-life (QOL), and disability post rTMS treatment (35).

When targeting the DLPFC with TMS, different repetitive or patterned stimulation protocols can be applied. In addition to the standard high frequency 10 Hz rTMS protocol administering 3,000 pulses in one of the in total 20–30 treatment sessions each lasting for ca 38 min (36), theta-burst stimulation (TBS) has more recently gained in popularity due to its much shorter treatment session duration. TBS mimics endogenous theta rhythms and has the ability to induce long-lasting effects on cortical excitability (37, 38). Intermittent TBS (iTBS) is one of the main patterns of TBS that have been developed, which increases cortical excitability (39), similar to high frequency 10 Hz rTMS but in a much shorter time frame. According to a recent systematic review and meta-analysis, TBS over DLPFC is well-tolerated and has significant antidepressant effects (40). A double-blind sham-controlled study of Li and colleagues among 60 treatment-refractory patients showed that iTBS is a safe, well-tolerated, and effective treatment for TRD (38). A large non-inferiority trial further indicated that iTBS has the same level of clinical efficacy as standard high frequency 10 Hz rTMS, thus offering a potentially much shorter and therefore cost-effective rTMS protocol alternative for TRD (41). In 2018, based on this study, FDA cleared the iTBS protocol for the treatment of MDD, in adult patients who have failed to receive satisfactory improvement from prior antidepressant medication.

A few case reports and a long-term retrospective review reported that the combination of ketamine and rTMS may be an effective long-term therapy for patients with depression (42–44). To the best of our knowledge, there is no study comparing the effectiveness of ketamine and rTMS in patients with depression in a naturalistic setting. Only a limited number of alternative non-pharmacological treatments for TRD are available today and more research is needed to directly compare a non-pharmacological treatment with a pharmacological treatment in terms of their efficacy and tolerability. In this study, we exploratively describe and compare the acute antidepressive efficacy of both, 8 sessions of intramuscular ketamine administered twice weekly for 4 weeks, as well as 30 sessions of left DLPFC-iTBS (over a period of 6 weeks) in depression patients seeking help in a naturalistic clinical mental health setting. While the iTBS protocol is FDA approved and by now a widely used method for the treatment of TRD in clinical practice, the potential use of IM ketamine in TRD has not been extensively researched and therefore is not widely used. This comparative study is important in order to point out that more research need to be done in this area and in order IM ketamine to be considered for FDA approval for TRD. Thus, the present study aimed to indicate for first time the potential of IM ketamine to reach similar effects in TRD as rTMS in shorter duration (less visits).

## Materials and Methods

### Design

A retrospective comparative study was conducted which included clinical records of TRD patients, as collected from the clinical database of Cyprus rTMS Center. The authors assert that all procedures contributing to this work comply with the ethical standards of the relevant national and institutional committees on human experimentation and with the Helsinki Declaration of 1975, as revised in 2008. All procedures involving human subjects/patients were approved by Cyprus National Bioethics Committee (EEBK E Π 2021.01.149) and written informed consent was obtained from all patients.

### Patients

Clinical records of twenty-four patients with treatment resistant depression who were referred to the Cyprus rTMS Center in the period of January 2018 to August 2021 and received either IM ketamine or rTMS as treatment for depression were included in this retrospective comparative study. During the clinical evaluation for treatment purposes, all patients were assessed using the ICD- 10 Classification of Mental and Behavioral Disorders and met the criteria for either moderate depressive episode or severe depressive episode without psychotic symptoms. All patients were on psychotropic medication (such as Sertraline and Venlafaxine) before, during and after the study. The Cyprus rTMS Center commonly offers both treatment options, IM ketamine and rTMS, to the patients. Treatment options were discussed with patients and literature findings were explained to them. Then, patients chose the treatment option (IM ketamine or rTMS) based on their preference. Twelve patients were treated with IM ketamine and twelve patients were treated with rTMS therapy using the iTBS protocol. An experienced psychiatrist and a TMS technician performed the rTMS treatment. Patients were reviewed regularly by the treating psychiatrist, every few weeks. The psychiatrist had regular contact with patients, weekly during the sessions of rTMS or ketamine, as well as a formal monthly review. Depression and anxiety severity were measured prior and after the completion of each treatment using clinician-rated and self-rated assessments (HDRS, HAM-A, BDI-II). The time between the two assessments (pre and post treatment) was not the same for both groups, as IM ketamine treatment was completed after 4 weeks and rTMS treatment was completed after 6 weeks. Patients thereafter followed an individual treatment plan, which may or may not, include maintenance and there was no relapse in their mental state for the following 4 months based on psychiatric reviews, no formal questionnaires were given. The criteria for inclusion of patients' clinical records in the study were: (1) patients aged 18 years and older, (2) patients meeting the criteria for either moderate depressive episode or severe depressive episode without psychotic symptoms, (3) patients not experiencing any significant clinical improvement from at least two different methods of antidepressants and (4) the existence of completed clinical evaluations prior and post treatment. The exclusion criteria were: (1) patients aged younger than 18 years and (2) mental objects or implants in the brain, skull or near head (e.g., pacemakers, metal plates). Demographic (age and gender) and depression severity (duration of current episode, number of episodes, duration of depression, number of unsuccessful antidepressants tried in current episode) data were collected.

### Clinical Assessments

#### Hamilton Depression Rating Scale and Hamilton Anxiety Rating Scale

HDRS (45) and HAM-A (46) are the most widely used depression and anxiety assessment scales to be administered by clinicians in order to assess the severity of depressive and anxiety symptoms, respectively. HDRS consists of seventeen items whereas HAM-A consists of fourteen items and a total score in both instruments is calculated by summing the individual scores from each item. In HDRS, the total score range of 0–52, where 0–7 is generally accepted to be within the normal range and represent the absence or remission of depression, while a score of 20 or higher indicated at least moderate severity. In HAM-A, the total score range of 0–56, where scores <17 indicated mild severity, scores 18-24 mild to moderate severity and scores 25–30 moderate to severe anxiety.

#### Beck Depression Inventory II

BDI-II is a one of the most widely used multiple-choice self-reported instruments that designed to assess depression severity (47). It consists of 21 items and the score of each item range from 0 to 3. The total score range of 0–63 with higher total scores indicating more severe depressive symptoms. Specifically, scores 0–13 indicated minimal range, scores 14–19 mild severity, scores 20–28 moderate severity, and scores 29–63 indicated severe depression.

### Treatment Procedure

As mentioned above, data of both experimental samples were retrospectively obtained from a real-life clinic. The patients had chosen the treatment method based on their preference; hence they were not randomly placed to these two experimental groups. However, both groups were being compared for relevant parameters (age, gender, depression severity) to ensure that they are not fundamentally different. Essentially, the only difference between the two experimental groups was the treatment method that they had received.

In the *rTMS treatment condition*, stimulation was performed using a MagPro X100 stimulator (MagVenture, Farum, Denmark) and a figure-of-eight coil (Cool-B65). Prior to stimulation, the individual resting Motor Threshold (rMT) was estimated over the left primary motor cortex (Mean = 50.25, SD = 4.03). The rMT is the amount of machine output (intensity) required to elicit a motor-evoked potential (MEP) in at least 50% of all attempts (48). Five iTBS sessions were administrated per week for 6 weeks, over the left DLPFC. To localize left DLPF, the software Beam_F3 Locator, an efficient and accurate method to mark the F3 position according to the 10-20 EEG system was used (49). Stimulation intensity was set at 120% of the rMT. The stimulation coil was placed at a 45° angle off the midline. iTBS was administrated at 5 Hz and each session included 20 trains with 8 s inter train interval (triplets of 50 Hz). A total number of 600 pulses was given per session for 3:08 min (41).

In the *ketamine treatment condition*, intramuscular ketamine was administrated twice weekly for 8 sessions. In the first session, patients received a dose of 0.25 mg/kg, and then the dosage was titrated upwards, to a maximum of 1 mg/kg by session 4, depending on patient effect and safe vital sign assessments in order to achieve the maximal antidepressant effect. All the necessary requirements were followed: ketamine was administrated by an experienced physician, the patient was monitored for 2 h after the administration under control settings and any side effects were recorded. The administration took place in a private room specially designed for the purposes of the treatment.

### Data Analysis

SPSS software version 27.0 was used for statistical analysis of data (IBM corporation, Endicott, New York). Independent sample *t*-tests and chi-square tests were used to compare the demographic and clinical characteristics between ketamine group and rTMS group. Due to the small sample size, Wilcoxon Signed-Ranks tests were used to evaluate changes in HDRS, HAM-A, BDI-II scores from pre treatment to post treatment for each experimental group individually and for the overall sample. The χ^2^ test was used to compare responders and remitters between the two groups. Responders were defined as patients with a 50% or greater decrease on the post treatment scores from the pre-treatment scores and remitters were defined as patients with HDRS post score ≤7, HAM-A ≤7 and BDI-II ≤13 (50–52). Mixed factorial ANOVAs were conducted to investigate the effect of both the within factor (Time) and the between factor (Experimental group). The within factor evaluated time depended effects (baseline vs. end of the treatment) on depressive and anxiety symptoms (HDRS, HAM-A, BDI-II). The between factor determined whether the patients who received ketamine had a different response compared with patients who received rTMS. The significance level was set at *p* < 0.05.

## Results

### Demographic and Clinical Characteristics

The clinical records of twenty-four TRD patients (11 male, mean age 47.9 ± SD 12.7) were collected. From these reports two groups were created, one group which received ketamine therapy and one group which received iTBS therapy. Analysis showed that both groups did not differ in demographic (age, gender) as well as clinical (duration of current episode, number of episodes, duration of depression, number of unsuccessful antidepressants tried in current episode, HDRS, HAM-A, BDI-II) characteristics. Accordingly, no significant differences were observed between the TRD patients who underwent the intramuscular ketamine therapy and those patients receiving rTMS (all *p* > 0.05; Table 1).

**Table 1:** Baseline characteristics of (*N* = 24) participants.

**Factors**	**Ketamine group**	**rTMS Group**	***df* values**	***p*-values**
	***n* = 12**	***n* = 12**		
**Demographic characteristics**				
Age (years)	44.08 (13.18)	51.67 (11.39)	22	0.146^a^
Gender (male/female)	5/7	6/6	22	0.682^b^
**Clinical characteristics**				
Duration of current episode (months)	5.50 (0.52)	5.67 (0.49)	22	0.430^a^
Number of episodes	3.08 (0.90)	3.58 (0.67)	22	0.137^a^
Duration of depression (years)	7.25 (3.70)	6.83 (3.01)	22	0.765^a^
Number of unsuccessful antidepressants tried in current episode	2.67 (0.78)	2.50 (0.80)	22	0.610^a^
HDRS Pre	32.33 (6.00)	30.25 (3.14)	22	0.298^a^
HAM-A Pre	34.83 (5.78)	34.17 (5.80)	22	0.781^a^
BDI-II Pre	45.25 (10.98)	38.67 (13.65)	22	0.206^a^

*Data are presented as the mean (with standard deviation, SD)*.

a *Independent sample t-tests*.

b *χ^2^ test*.

*rTMS, repetitive Transcranial Magnetic Stimulation; HDRS Pre, Hamilton depression rating scale before treatment; HAM-A Pre, Hamilton anxiety rating scale before treatment; BDI-II Pre, Beck depression inventory – II before treatment*.

### Treatment Outcomes

In the Ketamine group, a Wilcoxon Signed-Ranks test indicated that the post HDRSscores were significantly reduced compared to pre-treatment scores (Mean change = 26.08, SD = 7.33) (Z = −3.06, *p* < 0.005). Alike, post HAM-A scores were significantly reduced compared to baseline scores (Mean change = 29.08, SD = 6.93) (Z = −3.06, *p* < 0.005). Finally, significant reductions were observed also in BDI-II scores (Mean change = 32.50, SD = 15.40) (Z = −2.98, *p* < 0.005).

In the rTMS group, a Wilcoxon Signed-Ranks test indicated that post HDRS scores were significantly reduced compared to pre-treatment scores (Mean change = 23.18, SD = 3.97) (Z = −2.94, *p* < 0.005). Similarly, post HAM-A scores were significantly reduced compared to baseline scores (Mean change = 27.42, SD = 8.99) (Z = −3.06, *p* < 0.005). Finally, significant reductions were observed also in BDI-II scores (Mean change = 30.00, SD = 17.01) (Z = −2.93, *p* < 0.005).

### Response and Remission

Responders were defined as patients with a 50% or greater decrease from the baseline scores to the post treatment scores and remitters were defined as patients with HDRS post score ≤ 7, HAM-A ≤ 7, and BDI-II ≤ 13.

Out of a total of 12 patients in the Ketamine group, based on HDRS, 4 patients were responders (33.30%) and 8 patients were remitters (66.7%). Based on the HAM-A, the Ketamine group consisted of 3 responders (25%) and 9 remitters (75%). Finally, based on the BDI-II, 3 patients were responders (25%), 7 patients achieved remission (58.30%), whereas 2 patients were non-responders (16.70%) (Table 2).

**Table 2:** Responders and remitters, *n* (%).

		**Ketamine group**	**rTMS group**	***p*-values**
HDRS				0.565^a^
	Responders	4 (33.30%)	3 (25%)	
	Remitters	8 (66.70%)	8 (66.70%)	
	No responders	0 (0%)	1 (8.30%)	
HAM-A				1.00^a^
	Responders	3 (25%)	3 (25%)	
	Remitters	9 (75%)	9 (75%)	
	No responders	0 (0%)	0 (0%)	
BDI-II				0.535^a^
	Responders	3 (25%)	1 (8.30%)	
	Remitters	7 (58.30%)	9 (75%)	
	No responders	2 (16.70%)	2 (16.70%)	

a *χ^2^ test*.

*rTMS, repetitive Transcranial Magnetic Stimulation; HDRS, Hamilton depression rating scale; HAM-A, Hamilton anxiety rating scale; BDI-II, Beck depression inventory – II*.

Out of a total of 12 patients in rTMS group, based on HDRS, 3 were responders (25%), 8 achieved remission (66.70%), whereas 1 was a non-responder (8.30%). Based on the HAM-A, 3 patients were responders (25%) and 9 patients were remitters (75%). Finally, based on the BDI-II, 1 patient was a responder (8.30%), 9 patients achieved remission (75%), and 2 patients were non-responders (16.70%) (Table 2).

Overall, using χ^2^ tests, no significant differences were observed between the MDD patients of ketamine group and rTMS group in terms of responders, remitters and no-responders (all *p* > 0.05).

### Ketamine vs. RTMS

**Figure 1:**
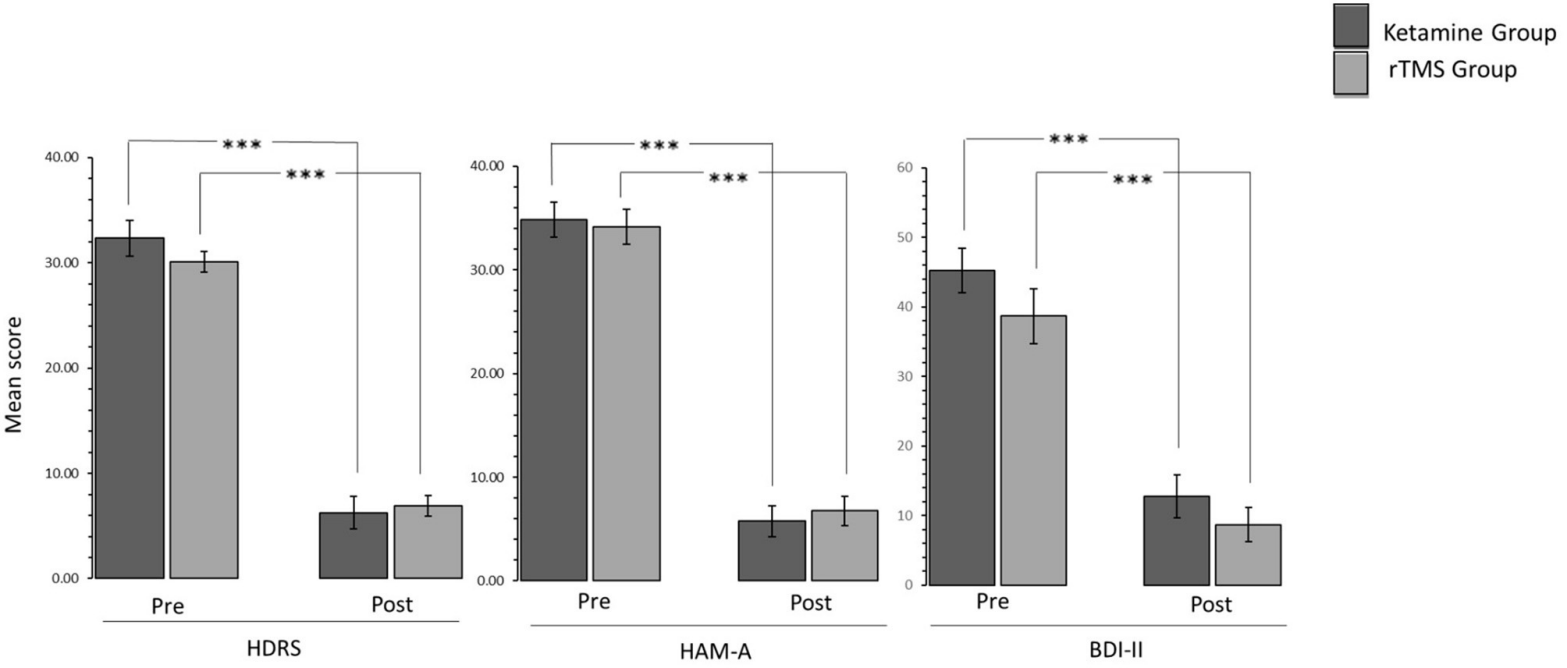
Bar graphs showing difference in pre-treatment and post-treatment scores of patients in Ketamine and rTMS groups. Error bars indicate standard error. ***P < 0.001.

2 (Time: pre-treatment, post-treatment) ^*^ 2 (Experimental Group: Ketamine Group, rTMS group) mixed factorial ANOVAs were conducted as measured by the three clinical assessments (HDRS, HAM-A, BDI-II). Results were consistent in all three clinical assessments. The interaction effect between Time and Experimental Group was not statistically significant [HDRS: *F*_(1,21)_= 1.355, *p* > 0.05, $\eta_{p}^{2}$ = 0.061; HAM-A: *F*_(1,22)_ = 0.258, *p* > 0.05, $\eta_{p}^{2}$ = 0.012; BDI-II: *F*_(1,22)_ = 0.142, *p* > 0.05, $\eta_{p}^{2}$ = 0.006]. There was a statistically significant main effect of Time [HDRS: *F*_(1,21)_ = 390.771, *p* < 0.05, $\eta_{p}^{2}$ = 0.949; HAM-A: *F*_(1,22)_ = 295.945, *p* < 0.05, $\eta_{p}^{2}$ = 0.931; BDI-II: *F*_(1,22)_ = 89.008, *p* < 0.05, $\eta_{p}^{2}$ = 0.802], suggesting a difference in the pre-treatment compared to post treatment. However, there was no significant effect of Experimental Group [HDRS: *F*_(1,21)_ = 0.273, *p* > 0.05, $\eta_{p}^{2}$ = 0.013; HAM-A: *F*_(1,22)_ = 0.013, *p* > 0.05, $\eta_{p}^{2}$ = 0.001; BDI-II: *F*_(1,22)_ = 2.934, *p* > 0.05, $\eta_{p}^{2}$ = 0.118]. Wilcoxon Signed-Ranks tests indicated that post HDRS (Z = −4.20, *p* < 0.005), HAM-A (Z = −4.29, *p* < 0.005) and BDI-II (Z = −4.17, *p* < 0.005) scores were significantly reduced compared to pre-treatment scores (Figure 1).

*Post-hoc* power analysis was conducted using the Superpower's Power Shiny App. Results showed that with 12 participants per group, we have 100% power for the main effect of Time. Also, the observed power of the main effect of Time was 1.00.

## Discussion

To our knowledge, this is the first study describing the effectiveness of both, ketamine treatment and rTMS treatment, in depressive and anxiety symptoms of MDD patients in a naturalistic real-life setting. Patients in the rTMS treatment group received 30 iTBS sessions over a period of 6 weeks, whereas patients in the Ketamine treatment group received 8 IM injections over a period of 4 weeks. Using three clinical assessments (HDRS, HAM-A, BDI-II), our data reveals that both therapies led to significant improvement in symptoms from pre- to post- treatment. Based on the HDRS, in the Ketamine group, 33.3% were responders and 66.7% were remitters and in rTMS group, 25% were responders and 66.7% were remitters. Based on HAM-A, in both experimental groups, 25% were responders and 75% were remitters. Finally, based on BDI-II, in Ketamine group, 25% were responders and 58.3% were remitters and in rTMS group 8.3% were responders and 75% were remitters. An explorative *post-hoc* direct statistical comparison indicated that ketamine therapy did not differ significantly from rTMS therapy with respect to pre- to post- depressive and anxiety symptoms, indicating that the effect of both experimental groups in our sample was equally effective. In line with this notion, statistical χ^2^ tests showed that there were no statistical differences between the patients of ketamine group and rTMS group in remission and response rates. These results indicated that IM ketamine therapy has the potential to reach similar effects in patients with TRD as rTMS therapy in a shorter treatment period as less visits are needed to complete the treatment. No significant side effects were reported from either the rTMS group or the ketamine group.

The results support the preliminary effectiveness of the treatments and adds to the existing literature regarding the efficacy of both treatment options in depression. Regarding TMS, a prior study by O'Reardon et al. (36), found that TMS was effective in treating MDD with minimal side effects. Furthermore, iTBS protocol, has proven to be an effective, safe and well-tolerated treatment for depression (38, 40, 41). Although there are many studies regarding the efficacy of ketamine in depression (5, 7, 11), the research in IM ketamine remains limited. There are only a few case reports that demonstrated the potential effectiveness of IM ketamine in depression, therefore the optimal use of IM ketamine warrants further investigation. A report on two cases with acute depression has shown that IM ketamine injection bring rapid relief from depressive symptoms and especially in the suicidal ideation (17). Another case report demonstrated that IM ketamine is a potential treatment for treatment-resistant bipolar depression (16). IM ketamine injection was also used in a female patient with metastatic ovarian cancer. The treatment was well-tolerated and after 6 sessions the patient achieved remission of her depressive symptoms (18).

Previous studies investigated the potential efficacy of combining ketamine and rTMS therapy in depression and bipolar disorder. However, to our knowledge, only a few case reports and a long-term retrospective review were reported so far. It is important to note that, a case report by Best and Grifflin, indicated that the combination therapy of ketamine and rTMS may be a more effective treatment for refractory depression, than either ketamine or rTMS alone (43). Furthermore, a recent long-term retrospective review demonstrated statistically significant reduction of depressive symptoms, after the combination therapy showing clear indication of the effectiveness of the treatment for refractory depression (44). Their review also found that this reduction in depressive symptoms could be sustained for a period of 2 years (44). Finally, according to some case reports, the combination therapy can be effective in treating severe depression in bipolar I disorder (42) and in bipolar II disorder (53).

Whereas previous research suggests that a combined treatment by ketamine and rTMS is an effective and long-term treatment for depression, the present comparative study represents a first attempt to describe and exploratively compare both treatment options as standalone therapies in a naturalistic setting. It is important to consider the limitations of our conclusions here. The current study is a retrospective comparative study with no a priori randomization and a very limited number of patients. Small sample sizes usually undermine the internal and external validity of a study and affect the generalizability of the results (54, 55). Especially for statistically comparing the effectiveness of two treatment options (clinical inferiority trial), a much larger same size would be needed. Another main limitation is the retrospective design of the study. Specifically, this study was based on data of patients with MDD, who were referred to the Cyprus rTMS Center in the past and received either intramuscular ketamine or rTMS as treatment for depression. Therefore, the patients were not randomly divided into these two experimental groups and no sham control groups were used. Finally, this study suffers from sample selection bias. A larger number of patients was treated with either IM ketamine or rTMS in the Cyprus rTMS center during that period, but we chose to include only patients who completed the total number of sessions required (rTMS: 30 sessions; Ketamine: 8 sessions) and patients with completed clinical evaluations prior and post treatment in our analysis. Unfortunately, we did not collect information about the number of patients with incomplete clinical evaluations prior or post treatment and the number of patients who terminated treatment prematurely. Thus, we selected only completers from a larger sample of patients of unknown size. Despite these limitations, this study could serve as a starting point for identifying and comparing the efficacy of these two depression treatments in a real life clinical setting.

Future research should further develop and confirm these initial findings by comparing the efficacy of ketamine treatment, rTMS treatment and the combination treatment in depression using a randomized, double-blind, sham-controlled clinical trial sufficiently powered to also reveal potential non-inferiority. Furthermore, clinical assessments should be collected weekly in order to investigate whether there are differences in response time between the treatment groups. In a future study, a follow up measurement is needed to examine and compare the long-term efficacy of these treatments. To the best of our knowledge, this comparative study was the first that directly compare the efficacy of rTMS and IM ketamine, a non-pharmacological treatment, and a pharmacological treatment for TRD. Finally, our results showed that the iTBS protocol, which has received FDA approval for MDD, and IM ketamine, which is not an FDA approved treatment for MDD, are equally effective treatments. This is an important finding as IM ketamine treatment is not widely used in clinical practice and can be administrated in a shorter duration compared to rTMS. Further research with more focus on the use of IM ketamine treatment in depression is therefore suggested, which may allow this treatment to gain a formal approval and a wider acceptance in daily practice.

## Conclusion

This retrospective study compared the efficiency of IM ketamine administered twice weekly for 8 sessions and 30 sessions of iTBS applied to the left DLPFC in MDD patients. Our results indicated significant pre- to post-treatment reduction in depressive and anxiety symptoms, with no significant differences between the two experimental groups, indicating that the effect of both therapies was equally effective in our limited sample. In line with this notion, response and remission rates were not statistically different between the two treatment groups. This study can be seen as a first step toward enhancing our knowledge regarding the therapeutic efficacy of two alternative depression treatment options such as ketamine therapy and rTMS therapy in a naturalistic real-life setting.

## Data Availability Statement

The original contributions presented in the study are included in the article/supplementary material, further inquiries can be directed to the corresponding author.

## Ethics Statement

The studies involving human participants were reviewed and approved by Cyprus National Bioethics Committee. The patients/participants provided their written informed consent to participate in this study.

## Author Contributions

GM, PM, and LP analyzed data and wrote the manuscript. TS and AS critically reviewed the manuscript and supervised the project. All authors agree with the contents of the manuscript and were fully involved in the study, preparation of the manuscript, have read the final version of the manuscript, and approved the submission.

## Conflict of Interest

The authors declare that the research was conducted in the absence of any commercial or financial relationships that could be construed as a potential conflict of interest.

## Publisher's Note

All claims expressed in this article are solely those of the authors and do not necessarily represent those of their affiliated organizations, or those of the publisher, the editors and the reviewers. Any product that may be evaluated in this article, or claim that may be made by its manufacturer, is not guaranteed or endorsed by the publisher.
